# Hearing Threshold Estimation With Distortion Product Otoacoustic Emission Growth Functions in People With Intellectual Disabilities in an Outreach Setting

**DOI:** 10.1111/jir.70043

**Published:** 2025-09-21

**Authors:** Eleftherios Savvas, Emmanouela Dimitrakopoulou, Harald A. Euler, Frans Coninx, Felix Bluemel, Anne Krieger, Karolin Schaefer, Philipp Mathmann, Corinna Gietmann, Katrin Neumann

**Affiliations:** ^1^ Department of Phoniatrics and Pedaudiology University of Muenster Muenster Germany; ^2^ Department of Otorhinolaryngology, Head and Neck Surgery, Division of Phoniatrics and Pediatric Audiology, St. Elisabeth‐Hospital Ruhr University Bochum Bochum Germany; ^3^ Department of Special Education and Rehabilitation, Faculty of Human Sciences University of Cologne Cologne Germany; ^4^ IfAP, Institut für Audiopädagogik Solingen Germany; ^5^ Institute for Special Needs Education (d/Deaf and Hard of Hearing) University of Duisburg‐Essen Germany

**Keywords:** audiometry, distortion product otoacoustic emission, DPOAE growth functions, hearing loss, hearing screening, intellectual disability

## Abstract

**Background:**

People with intellectual disabilities often have undetected hearing loss.

**Methods:**

In this prospective observational study featuring 110 adults with intellectual disabilities, hearing evaluations based on objective hearing threshold estimation using distortion‐product otoacoustic emission growth functions (DPOAEgfs) were performed in a sheltered workshop. Results were compared with thresholds obtained by conventional subjective pure‐tone audiometry (cPTA) and the adaptive self‐test PTA Multiple‐Choice‐Auditory‐Graphical‐Interactive‐Check (MAGIC).

**Results:**

The differences between the lowest thresholds obtained by cPTA and MAGIC for the frequencies 1, 2, 4 and 6 kHz (LPTATs) and the estimated distortion‐product thresholds (EDPTs) were calculated. Four hundred twenty‐seven of 880 (48.5%) pairs of measurements did not differ by any more than 5 dB. With LPTATs as reference criterion, for a favourable four‐frequency average over 1.0, 2.0, 4.0 and 6.0 kHz with a cutoff of 20 dB HL, the sensitivity of the EDPTs was 92% and the specificity 62%; a cutoff of 30 dB HL increased the respective values to 98% and 77%.

**Conclusions:**

DPOAEgfs are acceptable for estimating hearing thresholds in individuals with intellectual disability who may have limitations in performing conventional audiometry.

## 1 Introduction

The adequate medical care of people with intellectual disability is a major ongoing social and political issue. The care and support of this population entail teamwork between multiple professionals who provide proper support, skills training and education in order for them to be participating members of society. The prevalence of hearing impairment in this population is high, ranging from 6% to 55% depending on age and cause of intellectual disability (Erickson and Quick 2017; Evenhuis et al. 2003; Hild et al. 2008; Hildmann et al. 2002). Many genetic disorders known to cause intellectual disability are also associated with hearing loss in children, while others may cause sensorineural hearing loss related to early aging of hearing (Hildmann et al. 2002).

Hearing loss makes communication difficult for any individual affected; however, in people with intellectual disabilities, the effects are even more serious because of underlying issues around social behaviour, psychological status and neurocognitive function. Individuals often cannot express, or do not realise, that they have a hearing loss. Their ability to seek medical attention is therefore compromised. Even individuals who have been diagnosed with hearing loss are often lost to follow‐up before treatment can begin (Neumann et al. 2009). When intellectual disability and hearing loss are combined, the outcome is ‘complicating’ rather than ‘additive’ (Wiley and Moeller 2007) because the presence of a disability reduces the potential for compensation of other disorders (Erickson and Quick 2017; Knoors and Vervloed 2011). One reason for the high proportion of undiagnosed and untreated hearing loss may be the practical and organisational difficulties associated with travelling to a medical facility. A third person—an educator, caregiver or parent—must usually accompany the person to be tested, and special transportation may be required for individuals with additional significant motor disorders. The development and implementation of programmes for regular audiometric screening and diagnostics in the living environment of people with intellectual disabilities is therefore an important goal for better healthcare for this underserved population.

The study presented here is part of a larger prospective cohort study *Hearing Evaluation for Intellectual Disability* (HEID), which aimed to investigate various otological and audiometric methods used in a hearing screening and diagnostic programme for people with intellectual disabilities in their living environment. The goal of the present study is to find a valid and reliable method for estimating hearing threshold(s) in individuals with intellectual disabilities outside of standard clinical settings.

It is important to have a portfolio of several audiometric methods available in order to be able to combine individually adapted subjective and objective procedures for obtaining sufficiently reliable audiometric results. This is especially valuable within the context of intellectual disability: The varying severity levels and underlying traits of individuals affected lead to greater variability in the reliability of auditory thresholds obtained.

Pure tone audiometry (PTA) is one such test method and is reliable in most people with intellectual disabilities from a developmental age of 4 years onwards (Evenhuis et al. 2003; Hild et al. 2008). This test involves establishing a cooperative individual's subjective hearing responses to tonal stimuli, with the resulting auditory thresholds being represented graphically in an audiogram. However, methods of hearing threshold estimation should also be feasible where no active cooperation is possible. Such test methods would have been necessary in the larger HEID study in 5 of 120 participants (4.2%) who were unable to perform PTA.

Distortion‐product otoacoustic emission (DPOAE) testing is an objective method that does not require active cooperation and response by the test subject. DPOAE measures acoustic emissions from the inner ear that are produced when the cochlea is stimulated simultaneously by two pure tones with a frequency ratio between 1.1 and 1.3. DPOAE information can be represented in DP‐gram modality or as DPOAE growth functions (DPOAEgfs). DG‐grams measuring the DPOAE amplitudes arising at frequency 2*f*_1_–*f*_2_ are performed at different *f*_2_ frequencies. Stimulus intensities (*L*_1_ and *L*_2_) are fixed, for example, *L*_1_ = 65 dB SPL and *L*_2_ = 55 dB SPL. DG‐grams allow for a frequency‐specific assessment of cochlear function but not threshold estimation.

DPOAEgf does allow for the estimation of hearing thresholds (see Materials and Methods sections for more details). We hypothesised that DPOAEgf tests could be performed with an acceptable degree of accuracy even in a nonclinical setting, thereby enabling both screening use (e.g., when basic threshold information is required) and diagnostic application (e.g., to confirm or supplement other audiometric findings and to estimate hearing thresholds as precisely as possible) in cases where thresholds cannot be obtained through subjective audiometry. DPOAEgfs have been investigated in laboratory and some clinical settings, but their applicability for broader routine use has not yet been sufficiently explored in people with intellectual disabilities.

The present study aims to test the feasibility and validity of automated DPOAEgf measurements obtained in a mobile setting in persons with intellectual disability as an alternative or in addition to PTA procedures.

## 2 Materials and Methods

### 2.1 Participants

Potential participants with intellectual disability (128 adults, 58 women; mean age 40.8 years, range 19–62 years) who worked in sheltered workshops within a region of north‐western Germany were recruited for the HEID study. Individuals were included if undertaking hearing tests did not pose a risk to themselves or the examiners. Measurements were conducted with 120 participants (8 dropped out prior to beginning the tests due to a lack of interest). Complete data sets were obtained from 110 of these 120 participants. The individual reasons for dropouts and incomplete measurements are shown in Table 1.

**TABLE 1:** Dropouts among 128 participants and the following reasons for dropout: I = poor test compliance (e.g., participant walked around, was restless or spoke), II = unstable probe fit, III = test too difficult, IV = hearing loss > 80 dB HL (no data for numerical analysis), V = testing refused.

No. participant	cPTA	MAGIC	DPOAEgf
1	III		III
2	V	V	V
3	V	V	V
4		III	
5			II
6	V	V	V
7	V	V	V
8	V	V	V
9	V	V	V
10			II
11	III	III	
12	III	I	I
13	V	V	V
14	V	V	V
15		IIII	
16	III		
17		IV	
18	III		
**Total**	13 5 III, 8 V	13 1 I, 3 III, 1 IV, 8 V	12 1 I, 3 II, 8 V

All participants, or their parents or legal guardians, provided written informed consent for study participation. The information and consent form sent to participants was written in plain language and had been reviewed for comprehensibility by a committee of persons with intellectual disability. All procedures described in the clinical trial protocol followed the Good Clinical Practice guidelines and the Declaration of Helsinki (World Medical Association 2013). The study was approved by the Institutional Ethics Review Board of the University of Ruhr University Bochum (Approval Number 17–6186).

### 2.2 Setup of the HEID Study

The HEID study involved using mobile instrumentation to perform a range of audiometric tests commonly used in paediatric audiology, including a tympanometric evaluation of middle ear function. Otoscopy was also conducted and participants and workshop managers were asked about their history of known ear and hearing disorders. Participants' cognitive abilities were assessed using the Snijders–Ooomen Nonverbal Intelligence Test for ages 6–40 (SON‐R‐6‐40) (Tellegen et al. 2012). Auditory processing and language tests were also performed. All tests were administered by a neuroscience PhD student and two medical students (authors ED, FB and AK) who had received several days of training and supervision by paediatric audiologists. Complete testing took a maximum of 5 hours, spread over 5 days. Conventional PTA (cPTA) and the adaptive multiple‐choice‐auditory‐graphic‐interactive‐check (MAGIC) (Bohnert et al. 2010; Schirkonyer et al. 2010) yielded the lowest hearing thresholds in the HEID study and were therefore used as reference tests in the part of the study described here. These procedures will be detailed in the next section.

### 2.3 Procedure

The order of cPTA, MAGIC and DPOAEgf testing was deliberately not predetermined but was instead adapted to the individual cognitive and physical capacity of each participant. The feasibility of testing was documented for each participant and each session within the study protocol. If compliance was insufficient, testing was repeated on a different day. If adequate measurement quality still could not be achieved, the data were excluded from the analysis. Only data collected with sufficient quality are reported here. The total duration for these tests was a maximum of 1 hour. DPOAEs were recorded at *f*_2_‐frequencies of 1.0, 1.5, 2.0, 3.0, 4.0, 6.0 and 8.0 kHz. cPTA was performed at 0.5, 1.0, 2.0, 4.0 and 8.0 kHz; MAGIC was additionally performed at 6.0 kHz. Different frequencies were analysed using these tests for two reasons: 1) the optimal frequencies by which the lowest error rates in estimation of hearing thresholds by extrapolation had to be established (typically 2.0, 3.0 and 4.0 kHz, with an upper test limit of 6 kHz (Gorga et al. 2003)); 2) in order to maximise the attention of the participants, thereby increasing the reliability of the data obtained, the measurement times for PTA measurements (cPTA and MAGIC) had to be limited to what was absolutely necessary. All three tests were measured using a portable audiometric device, Sentiero (PATH MEDICAL GmbH). Sound‐attenuating circumaural headphones (HDA 300 by Sennheiser Electronic GmbH & Co., Wedemark, Germany) were used for the cPTA and MAGIC tests. DPOAEgfs were measured using an EP‐TY ear probe (SN: 40011, PATH MEDICAL GmbH). All test equipment was calibrated in accordance with the manufacturer's procedures and national medical device legislation at the beginning of the study.

Tests were performed in a quiet room within the sheltered workshop. Care was taken to ensure that ambient noise levels did not exceed 35 dB SPL (measured with a B&K 2250 Sound Level Meter/Analyser, Brüel & Kjaer; A‐weighted, fast time constant) in each of five frequency bands from 0.25 to 4 kHz.

MAGIC is an image‐based, self‐controlled, playful alternative to cPTA. Users select a picture of an animal on the touch display of the device to start an interactive game‐like workflow. Pressing again on the animal initiates the presentation of a tone, following which pictures of a healthy variant and an ill variant of the same animal are displayed. If the participant hears the tone, they should select the healthy‐looking animal; if they do not hear the tone, they should select the ill‐looking animal. The levels of subsequent tone presentations adapt according to the responses of the participant (‘not heard’ leads to subsequent presentation at a higher level, ‘heard’ leads to subsequent presentation at a lower level). Trials with no tonal presentation (‘no‐sound trials’) were used to assess false negatives and monitor attention. Such trials were included after every third heard stimulus, but not at the beginning of a new frequency block or after swapping between ears. Different animals represent different frequencies (e.g., the bear represents 0.5 kHz; the mouse 4.0 kHz). The validity and reliability of this test have been established (Bohnert et al. 2010; Schirkonyer et al. 2010).

DPOAEs are sound signals generated within the cochlea by stimulating with two primary sinusoidal tones that excite the basilar membrane at their best frequency sites and have a specific relationship between their frequencies *f*_1_ and *f*_2_. The ratio between the two frequencies *f*_2_/*f*_1_ used here was equal to 1.2 for all test conditions. The stimuli are delivered to the ear canal using an insert probe. In response to this stimulation, a third tone with a third frequency, the DPOAE, is generated by interaction in the overlapping basilar membrane region of the two travelling waves (often called the ‘distortion component’) and from energy that travels from the area of overlap to the 2*f*_1_–*f*_2_ frequency site (often referred to as the ‘reflection component’). The present study employed the most commonly used frequency for DPOAE measurements, *f*_DP_ = 2*f*_1_–*f*_2_. A 6 dB signal‐to‐noise ratio (SNR) criterion is typical when identifying a DPOAE as present (Hoth et al. 2015). In general, DPOAEs consist of the following two above‐mentioned components: the nonlinear distortion component and the coherent reflection component (Zelle et al. 2016). Both components propagate retrogradely toward the ear canal. Depending on the amplitude ratio and the relative difference in phase between the two DPOAE components, interference between the components occurs (Shera and Guinan 1999; Zelle et al. 2016). These biological interference signals cause periodic variation of the DPOAE amplitude known as the DPOAE fine structure in high resolution DP‐grams.

DPOAEgfs are obtained by measuring DPOAE amplitude as a function of stimulus level(s) with a fixed stimulus frequency combination (Kummer et al. 1998). Past work has suggested that DPOAEgfs may provide an objective estimate of pure‐tone thresholds (Boege and Janssen 2002). In this test method, the level *L*_2_ of the tone with the higher stimulus frequency *f*_2_ is set and *L*_1_ is adjusted according to the formula *L*_1_ = 0.4*L*_2_ + 39 dB (Kummer et al. 2000). A linear regression analysis is performed in order to approximate the relationship between the DPOAE pressure *p*_DP_ (in μPa) and the primary tone level *L*_2_ (range from 15 to 65 dB SPL) (Boege and Janssen 2002). The device starts with a step size of 20 dB, which is reduced to 10 dB and finally to 5 dB. It automatically attempts to obtain at least three valid OAE responses based on the built‐in SNR and minimum OAE level criteria. The extrapolation of the DPOAE pressure I/O‐function is performed in order to estimate the DPOAE threshold. The intersection point of the linear regression line with the *L*_2_‐axis at *p*_DP_ = 0 Pa serves as an estimate of the DPOAE threshold. This method seems to allow an estimation of the DPOAE threshold up to a hearing loss of 50 dB SPL (Boege and Janssen 2002; Gorga et al. 2003; Kummer et al. 2000). According to the manufacturer (PATH MEDICAL GmbH), the procedure for estimating hearing thresholds from extrapolated distortion product otoacoustic emission I/O functions as described above (proposed by Boege and Janssen 2002) has been implemented in the Sentiero device. The results as calculated by the Sentiero were used for analysis. Preset auto‐settings were selected for the measurements: primary tone settings *L*_2_/*L*_1_; frequency ratio *f*_2_/*f*_1_ = 1.2; adaptive timeout (which adapts the measurement duration of a given frequency and level in real‐time depending on noise and otoacoustic emission levels); and cubic distortion product 2*f*_1_–*f*_2_. Thresholds were estimated by linear regression analysis. The thresholds were determined exclusively by the software, not interpretation by the testers. The starting level for the threshold search was *L*_*2*_ = 65 dB SPL. The further test procedure was as described by Boege and Janssen (2002).

### 2.4 Statistical Analysis

Statistical analysis was performed using SPSS version 27.0 (SPSS Inc., Chicago, Illinois, USA) and RStudio (V.1.4.1106, R Core Team 2020).

In order to compare the hearing thresholds estimated using extrapolated DPOAEgf data—here known as estimated distortion‐product thresholds (EDPTs) for ease of reading—with the best available PTA reference, the lowest PTA thresholds for each of the frequencies 1.0, 2.0, 4.0 and 6.0 kHz were determined from the measurements of cPTA and MAGIC and implemented as a novel parameter ‘lowest PTA threshold’ (LPTAT). The above‐named frequencies capture both DPOAEgf and PTA in our protocol and reflect the main frequency range of speech, except for the low frequencies where DPOAEs are not measurable. In order to allow comparison between the LPTATs per frequency and the EDPTs, which are not ascertainable from hearing thresholds > 50 dB HL, LPTAT values of > 50 dB HL were set to 50 dB HL before their mean value per frequency was calculated. Frequency‐specific individual differences between LPTATs and EDPTs were then calculated.

Inferential statistics of the EDPT, LPTAT and of the cPTA and MAGIC thresholds across the frequencies analysed was performed with the Wilcoxon test for nonparametric data. Bonferroni correction was applied.

Receiver operating characteristic (ROC) curve analysis was also performed in order to calculate an optimal relationship of specificity and sensitivity of EDPTs for three cutoff thresholds of ≥ 20, ≥ 30 and ≥ 40 dB HL related to the LPTATs as reference (Junge and Dettori 2024). The discriminatory power of the model was considered excellent with an area under the curve (AUC) value of > 0.9, considerable with 0.8 ≤ AUC < 0.9, moderate with 0.7 ≤ AUC < 0.8 and poor with 0.6 ≤ AUC < 0.7 (Çorbacıoğlu and Aksel 2023).

## 3 Results

For all 110 participants included in the analysis, the results of the SON‐R‐6‐40 ranged between IQ scores of 55 and 92 (mean, 60.9). This indicated that all participants had a mild intellectual disability (IQ, 50–69) or better. Eleven participants had been assumed to have intellectual impairments but received IQ scores above 70. These 110 participants completed the DPOAEgf and PTA measurements as described in the Procedures section. The average time required to complete cPTA on both ears was 6:51 (mins:secs) (±2:57, 5 frequencies); 7:45 (±2:36, 6 frequencies) for MAGIC; and 9:18 (±5:20, 7 frequencies) for DPOAEgf.

**FIGURE 1:**
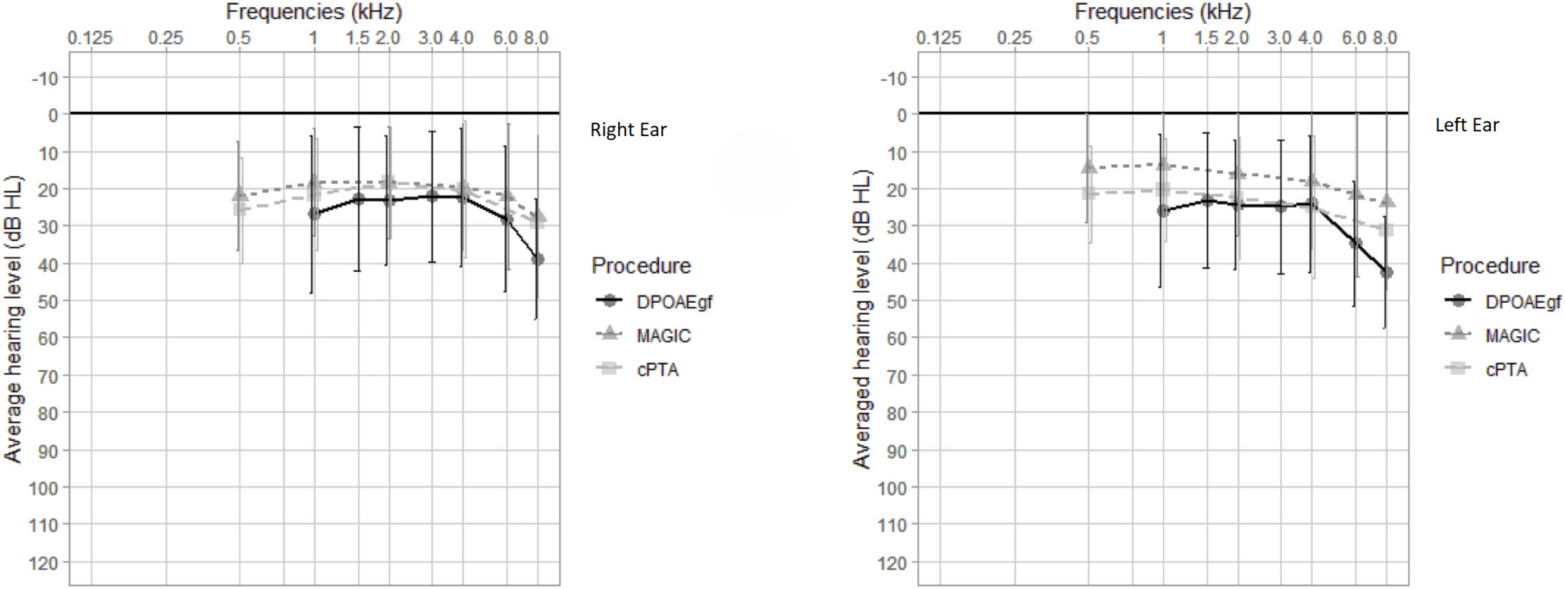
Averaged thresholds of MAGIC, cPTA and DPOAEgf for the right and left ears.

**FIGURE 2:**
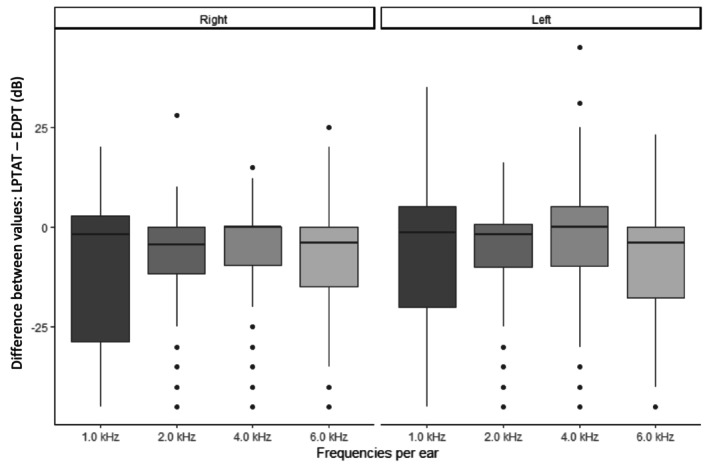
Boxplots of differences of LPTAT minus EDPT for single frequencies for the right and left ears. Lowest differences were obtained for 2.0 and 4.0 kHz.

The average thresholds for each of the frequencies of cPTA and MAGIC, and the respective EDPT and LPTAT values of all 220 ears are shown in Table 2; corresponding audiograms can be found in Figure 1. The mean EDPT averaged over all four frequencies was 26.2 dB HL (SD, 19.03), and the mean LPTAT was 18.99 dB HL (SD, 14.1). Boxplots of the frequency‐specific deviations of the EDPTs from the reference LPTATs are demonstrated in Figure 2.

**TABLE 2:** Mean thresholds of cPTA, MAGIC, LPTAT, DPOAEgf and LPTAT‐EDPT differences for 1.0, 2.0, 4.0, 6.0 kHz and for the averages over all frequencies, with standard deviation (SD) in parentheses.

Frequency (kHz)	cPTA (dB HL)	MAGIC (dB HL)	LPTAT (dB HL)	EDPT (dB SPL)	Difference LPTAT‐EDPT (dB)
1.0	20.98 (14.38)	18.91 (13.98)	16.95 (11.61)	26.45 (20.71)	−9.5 (17.64)
2.0	20.5 (15.83)	20.05 (15.72)	17.16 (13.16)	23.73 (17.36)	−6.57 (12.75)
4.0	22.61 (18.73)	22.16 (17.67)	18.55 (14.09)	23.22 (18.45)	−4.67 (14.32)
6.0	—	25.73 (20.97)	23.32 (16.1)	31.47 (18.42)	−8.15 (14.48)
**Overall**	**21.36 (16.42)**	**21.71 (17.45)**	**18.99 (14.1)**	**26.22 (19.03)**	**−7.23 (14.99)**

*Note:* To allow comparison between the LPTATs and the EDPTs, which are not ascertainable from hearing thresholds > 50 dB SPL, LPTAT values of > 50 dB HL were set to 50 dB HL. Therefore, and because the lowest values of cPTA and MAGIC were chosen for the calculation of the LPTAT, the latter values are lower than the average threshold values of cPTA and MAGIC.

We compared the average thresholds measured across the four frequencies 1.0, 2.0, 4.0 and 6.0 kHz of LPTATs (4fLPTAT) and EDPTs (4fEDPT) using a method adapted from the WHO's classification of grades of hearing loss (averaging PTA threshold values over the four frequencies 0.5, 1.0, 2.0 and 4.0 kHz [4fPTA] (World Health Organization, 2021)). It should be noted that the 6‐kHz criterion, as can be seen for example from the normative curves for age‐related hearing loss (Löhler et al. 2019), captures a greater proportion of hearing loss in the population than the 500‐Hz criterion.

The 4fLPTAT was 7.9 dB lower than the 4fEDPT for the right ears and 6.5 dB for the left ears. The Bonferroni‐corrected Wilcoxon test for nonparametric data reveals a significant difference between cPTA and MAGIC thresholds at 1.0 kHz (*p* < 0.001), but not at 2.0 kHz (*p* > 0.999) or 4.0 kHz (*p* > 0.999) across all 220 ears. For both cPTA and MAGIC thresholds, the analyses showed no significant deviations from EDPT measurements at 1.0 kHz (cPTA *p* = 0.013; MAGIC *p* < 0.001), 2.0 kHz (cPTA *p* = 0.02; MAGIC *p* = 0.019) and 6.0 kHz (*p* < 0.001; data available for MAGIC alone) but not at 4.0 kHz (cPTA *p* > 0.999; MAGIC *p* > 0.999). A significant difference was found for all comparisons for LPTAT and EDPT (all frequencies *p* < 0.001). According to the Wilcoxon test for nonparametric data, all three‐ or four‐frequency averages across 1.0, 2.0, 4.0 and 6.0 kHz (with 6.0 kHz excluded for cPTA) for cPTA, MAGIC and LPTAT differed significantly from the EDPTs (cPTA *p* = 0.004; MAGIC *p* < 0.001; LPTAT *p* < 0.001). Using the same Wilcoxon test, no significant difference was found between the cPTA and MAGIC thresholds averaged over 1.0, 2.0 and 4.0 kHz (*p* = 0.49).

From the total of 880 paired EDPT and LPTAT values (220 ears at 4 frequencies each), 427 pairs (48.5%) either matched exactly or differed by no more than ±5 dB (light yellow squares in Figure S1). In 123 of the 880 pairs (14%), the EDPTs exceeded the corresponding LPTATs by at least 30 dB (i.e., a potential hearing loss was markedly ‘overestimated’ when compared to the LPTATs, which served as the clinical gold standard for threshold assessment [dark green squares in Figure S1]). In contrast, only three of 880 pairs (0.3%) showed EDPT values of at least 30 dB lower than the LPTATs, i.e., a hearing loss was substantially ‘underestimated’ in three of 110 subjects, but only in one ear and one frequency per participant (dark‐red squares in Figure S1). EDPTs were 20–29 dB lower than LPTATs in another 10 participants (light red squares in Figure S1) but only in one of eight frequencies measured (10 ears). EDPTs were 10–19 dB lower than LPTATs in nine individuals (11 ears) (orange squares in Figure S1), at three of four frequencies for three of those individuals (three ears) and at two frequencies in six individuals (eight ears). LPTATs were lower by 10–19 dB in one frequency and by 20–29 dB in another frequency in each of six participants (six ears) (combination of light red and orange squares).

Eighty‐three (37.8%) of the 220 ears examined had a 4fLPTAT value of ≥ 20 dB HL, and 4fEDPT values met this criterion in 128 ears (58.2%). Average thresholds of ≥ 20 dB HL are indicative of hearing loss according to the WHO criteria (World Health Organization 2021), although there are some deviations in the frequency range compared to the LPTAT frequencies, as outlined above. In a small number of individuals (six individuals, each with one affected ear), the criterion for hearing loss (average hearing threshold ≥ 20 dB) was fulfilled according to 4fLPTATs but not 4fEDPTs. However, because their mean LPTATs in the respective ears exactly met the threshold of ≥ 20 dB or exceeded it only by a maximum of 8.75 dB, the potential oversight of such a hearing loss may be considered tolerable. Nonetheless, the overarching goal should remain the identification and management of every hearing loss that warrants intervention.

For all participants and ears, middle ear function was assessed using tympanometry. Of the 220 ears examined, 196 (89.1%) showed a normal tympanogram (Jerger Type A), 12 ears (5.4%) had a flat curve (Type B), indicating conditions such as otitis media with effusion, and 3 ears (1.4%) showed negative middle ear pressure (Type C). Tympanometry could not be performed for 9 ears (4.1%). In 14 of the 15 cases with Jerger type B or C tympanograms, we observed—consistently and often across multiple frequencies—that EDPTs exceeded the corresponding frequency‐specific LPTATs by 25–45 dB. In these cases, the degree of potential hearing loss appeared markedly overestimated when compared to the LPTAT results. Regardless, the combination of abnormal tympanometric findings and elevated EDPTs should have warranted referral for further diagnostic evaluation.

We determined the validity of the EDPT with reference to the LPTAT for our sample based on the average of the frequencies 1.0, 2.0, 4.0 and 6.0 kHz in order to discriminate between normal hearing and a potential hearing loss of ≥ 20 dB HL. The sensitivity related to ears, according to the above criterion, was 92% (95% confidence interval [CI], 83%–97%) and the specificity 62% (95% CI, 53%–70%). The positive predictive value of screening with this cutoff was 0.59 (95% CI, 0.5–0.68); the negative predictive value was 0.92 (95% CI, 0.85–0.97). If hearing loss were to be assumed only on the basis of a 4fLPTAT of ≥ 30 dB HL, the sensitivity would increase to 98% (95% CI, 0.87–1.00) and the specificity to 77% (95% CI, 0.7–0.83). For a cutoff criterion of ≥ 40 dB HL, the sensitivity would be 95% (95% CI, 0.75–1.00) and the specificity 83% (95% CI, 0.77–0.88). With PTA as the gold standard and the strict ≥ 20 dB HL cutoff criterion, 52 (47.3%) of participants (83 ears, 37.7%) would have been suspected of having a hearing loss (40 bilateral, 12 unilateral). Seventy‐three participants (66.4%) (128 ears, 58.2%; 55 bilateral, 18 unilateral) would have been su spected of HL using DPOAEgf.

**FIGURE 3:**
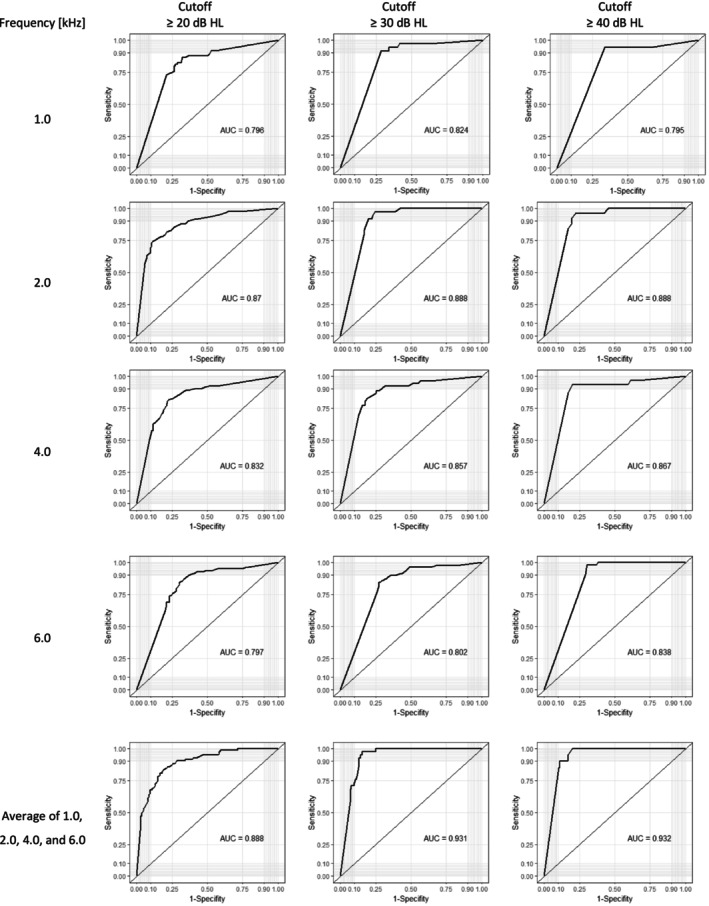
ROC curves and corresponding AUC values for 1.0, 2.0, 4.0 and 6.0 kHz and for their averages to visualise optimal fits of EDPT specificities and sensitivities for cutoff hearing thresholds at 20, 30 and 40 dB HL.

We performed ROC analyses using hearing threshold cutoffs at 20, 30 and 40 dB HL to evaluate the validity of using DPOAEgf as an indicator of hearing loss (DPOAEgf values shown here were already converted to dB HL using the algorithm integrated within the device). ROC curves were calculated for both the individual frequencies and for the average of their possible combinations. More precisely, LPTATs were used as the gold standard for the hearing loss to which the EDPT thresholds were compared in order to determine the ROC curves and AUC. The AUC for each frequency and the average of those frequencies are presented in Table 3. The corresponding ROCs and the sensitivity and 1‐specificity values that can be read from them are shown in Figure 3. The highest AUC values were obtained for the four‐frequency averages, with optimal sensitivity to specificity ratios of 81.8%–83.1% for the ≥ 20 dB HL, 97.6%–84.4% for the ≥ 30 dB HL and 100%–80% for the ≥ 40 dB HL cutoff criteria. For individual frequencies, the largest AUCs for all cutoff thresholds were determined at 2.0 kHz, followed by 4.0 kHz (Table 3, Figure 3).

**TABLE 3:** AUC for 1.0, 2.0, 4.0 and 6.0 kHz and for the averages over all frequencies of the ROC analyses with cutoff hearing thresholds at 20, 30 and 40 dB HL.

	Frequency (kHz)				
Cutoff threshold (dB HL)	1.0	2.0	4.0	6.0	Average over 1.0, 2.0, 4.0, 6.0
≥ 20	0.796	0.87	0.832	0.797	0.888
≥ 30	0.824	0.888	0.845	0.802	0.931
≥ 40	0.795	0.888	0.867	0.838	0.932

A Spearman rank correlation between 4fLPTAT and 4fEDPT was 0.76 (*p* < 0.001). A linear regression revealed an adjusted *R* ^2^ = 0.585. There was a moderate negative correlation between IQ and LPTAT *(Spearman's ρ* = −0.3, *p* = 0.001) but only a weak, nonsignificant correlation between IQ and EDPT (Spearman's *ρ* = −0.18, *p* = 0.06).

## 4 Discussion

This study aimed to answer the question of whether the use of DPOAEgfs to estimate hearing thresholds could be feasible and acceptably valid as part of an outreach mass hearing evaluation programme for people with intellectual disabilities within their living environment. This question could be answered in the affirmative, with some specific, acceptable limitations.

Measurements were feasible in 90.8% of participants who consented to hearing screening, with only 6.7% refusing further participation and 2.5% limited by unstable probe fitting. Dropout rates of 11%–12% were comparable for all methods. This high compliance indicates that the DPOAE measurement, despite the probe in the ear canal and the necessity to sit quietly, is well accepted and comparable to PTA (Table 1). In addition, the familiar environment, the proximity of caregivers who can act as mediators in communication, and the absence of transportation—often perceived as stressful—probably increase the participants' willingness to undergo hearing testing in their social environment rather than in a clinical setting.

Ample research has already been published on DPOAE as a screening method, for example, in the context of newborn hearing screening (e.g., Gorga et al. 2000; Janssen 2013; Mishra et al. 2013; Norton et al. 2000) or the Special Olympic games for people with intellectual disability (e.g., Kumar Sinha et al. 2008; Neumann et al. 2006). Here, however, we have moved beyond considering the DPOAE as a simple screening procedure to assessing its use as a threshold estimation method.

A moderate negative correlation was found between IQ scores and PTA thresholds, but no significant correlation between IQ and EDPT. This suggests that while behavioural thresholds (PTA) may partially relate to cognitive abilities, EDPT values appear to vary independently of IQ, thus indicating that additional factors may influence these objective measures. We had hypothesised that a correlation between PTA thresholds and severity of intellectual disability, operationalised as IQ score, would be found and that objective audiometric methods would be required in order to assess hearing thresholds in individuals with severe or moderate intellectual disability. However, because the study sample only included participants with mild intellectual disability or IQ within the normal range, this correlation could not be measured, and instead, PTA could be used as the reference standard for hearing thresholds. As a result, the findings cannot be generalised to individuals with more profound intellectual disability, for whom the reliability of behavioural audiometry remains uncertain.

PTA measurements indicated that 47% of participants met the hearing loss criterion of ≥ 20 dB HL, whereas DPOAEgf identified 66% as meeting this cutoff, suggesting a higher proportion of individuals who may require further audiological assessment. However, it is important to note that in the context of a large‐scale hearing evaluation programme, only a small subset of individuals would undergo DPOAEgf testing—specifically, those for whom PTA is not feasible or does not produce sufficiently reliable results.

The 92% sensitivity of the DPOAEgf method at the ≥ 20 dB criterion for hearing loss was high, meaning that it appears to be well adapted to the WHO 4fPTA hearing loss criterion of ≥ 20 dB HL (World Health Organization 2021). With DPOAEgf, only 3.2% of the 220 ears examined were incorrectly classified as having normal hearing (i.e., false negatives relative to PTA thresholds ≥ 20 dB HL); thus, if DPOAEgf had been used as the only screening method, 4 out of 110 individuals (3.6%) would not have been referred for necessary further audiological testing. In all of these cases, the hearing loss would have been only slightly underestimated by DPOAEgf, with the maximum deviation from the PTA threshold being 8.75 dB. The specificity of the DPOAEgf threshold estimation was only 62% for the ≥ 20 dB HL criterion. In other words, with this criterion approximately 19% more participants (20.5% of ears) would have needed to undergo detailed hearing diagnostics than if they had been tested with PTA alone. However, the relatively high false‐positive rate of DPOAEgf screening (in comparison with the PTA) seems tolerable given the high prevalence of hearing loss in adults with intellectual disabilities (approximately one‐third to one‐quarter (Hild et al. 2008; Neumann et al. 2006)) and the fact that in a planned German programme of hearing detection and intervention for people with intellectual disability, detailed audiological diagnostics would, in most cases, immediately follow failed hearing screening at the same institution (Schwarze et al. 2023). In this context, prioritising high sensitivity over specificity is the safer and more appropriate approach.

Based on the fact that the measurements were not made under audiometric conditions in a soundproof booth and that the participants had an intellectual disability, it makes sense to relax the criterion of hearing loss. If a 4fLPTAT of ≥ 30 dB HL criterion were to be implemented, the sensitivity would increase to 98% and the specificity to 77%; for a cutoff criterion of ≥ 40 dB HL, the sensitivity would be 95% and the specificity 83%. The AUC values of the ROCs, which allow optimal adjustment of sensitivity and specificity, point in the same direction. In addition, both cutoffs (30 and 40 dB HL) seem reasonably acceptable in light of the fact that the WHO (World Health Organization 2021) classified ‘disabling hearing loss’ as a permanent, unaided hearing loss in the better hearing ear (averaged over 0.5, 1.0, 2.0 and 4.0 kHz) of > 30 dB HL in children and > 40 dB HL in adults (Neumann et al. 2019; Olusanya et al. 2014). On this basis the authors recommend that within a mass screening and diagnostic programme, a DPOAEgf threshold of > 30 dB HL averaged over the frequencies 1.0, 2.0, 4.0 and 6.0 kHz should be regarded as a trustworthy indication of hearing loss because of the apparently optimal sensitivity–specificity ratio. If the seemingly more desirable lower cutoff value of ≥ 20 dB HL were used, less optimal specificity and especially sensitivity values would have to be accepted. Our results are consistent with the findings of Gorga et al. (1997), who reported that test performance—as defined by the AUC—was optimal when normal hearing was defined as audiometric thresholds between 20 and 30 dB HL. Performance was highest for mid and high frequencies, and poorest for low frequencies as well as for the highest frequency tested, 8.0 kHz.

The feasibility of DPOAEgf measurements within a hearing evaluation programme for persons with intellectual disability is also supported by their relatively short measurement duration. The mean duration per frequency for DPOAEgf falls within the same range as that of the PTA‐based methods (DPOAEgf: 79.7 s, cPTA: 82.2 s, MAGIC: 77.5 s).

One could reasonably question why alternative objective audiometric methods for estimating hearing thresholds were not favoured over DPOAEgf. Our planned programme of testing within the living environment and subsequent on‐site diagnostics, treatment and monitoring where necessary, of people with intellectual disabilities, includes measurements of transient‐evoked otoacoustic emissions (TEOAEs), DP‐grams, DPOAEgfs and auditory evoked potentials (AEPs). The advantage of the TEOAE is its short measurement time and simple execution; the disadvantage is its lower frequency specificity than DPOAE and its very limited suitability for determining hearing threshold. In contrast, DP‐grams have the advantage of frequency specificity and short measurement time, because only one stimulus level is measured, but huge variation in emission levels even among otologically normal individuals is expected (Mills et al. 2007). It is therefore impossible to predict a hearing threshold from one single emission level at one frequency. The measurement of frequency‐specific auditory evoked potentials or auditory steady‐state responses is time‐consuming and requires the application of scalp electrodes, among other factors, that complicate its use in an outreach setting with this population. DPOAEgf, on the other hand, combines the advantages of relative brevity, objectivity, frequency–specificity and acceptable diagnostic accuracy, as demonstrated by previous research (Gorga et al. 1997). Moreover, DPOAEgf yielded the lowest estimated hearing thresholds after the PTA procedures out of several subjective and objective audiometric procedures investigated within the HEID study. Though not ideal, DPOAEgf represents a (currently) good alternative in order to avoid the following, far less favourable, solutions in cases where PTA is not feasible: (a) abandoning testing and missing audiological information, (b) referring these individuals for further assessment in a clinical setting (occurs in approx. 2% of individuals (Neumann et al. 2009)) and (c) attempting to measure frequency‐specific auditory brainstem evoked responses or auditory steady‐state responses, which often have only mediocre accuracy in nonclinical settings.

Assessing the hearing status of people with intellectual disabilities requires a high degree of flexibility, i.e., screening and diagnostic procedures are closely interconnected and often applied together. The aim of such audiological assessment is to achieve the best possible approximation of the actual hearing threshold given the environmental restrictions, as people with intellectual disabilities are very rarely brought to clinics by their caregivers (Ali et al. 2013). The use of DPOAE recordings within a planned nationwide hearing screening and diagnostic programme for people with intellectual disabilities carried out in their living environment could be twofold: On the one hand, the DP‐grams can be obtained quickly, in approximately 2 min and may be useful for screening and plausibility checks of a mainly PTA‐based screening assessment (Gorga et al. 1993), with the DPOAgf being used for diagnostics when screening has been failed and further confirmation is required. On the other hand, DPOAEgf threshold screening may be required in cases where PTA screening is impossible.

**FIGURE 4:**
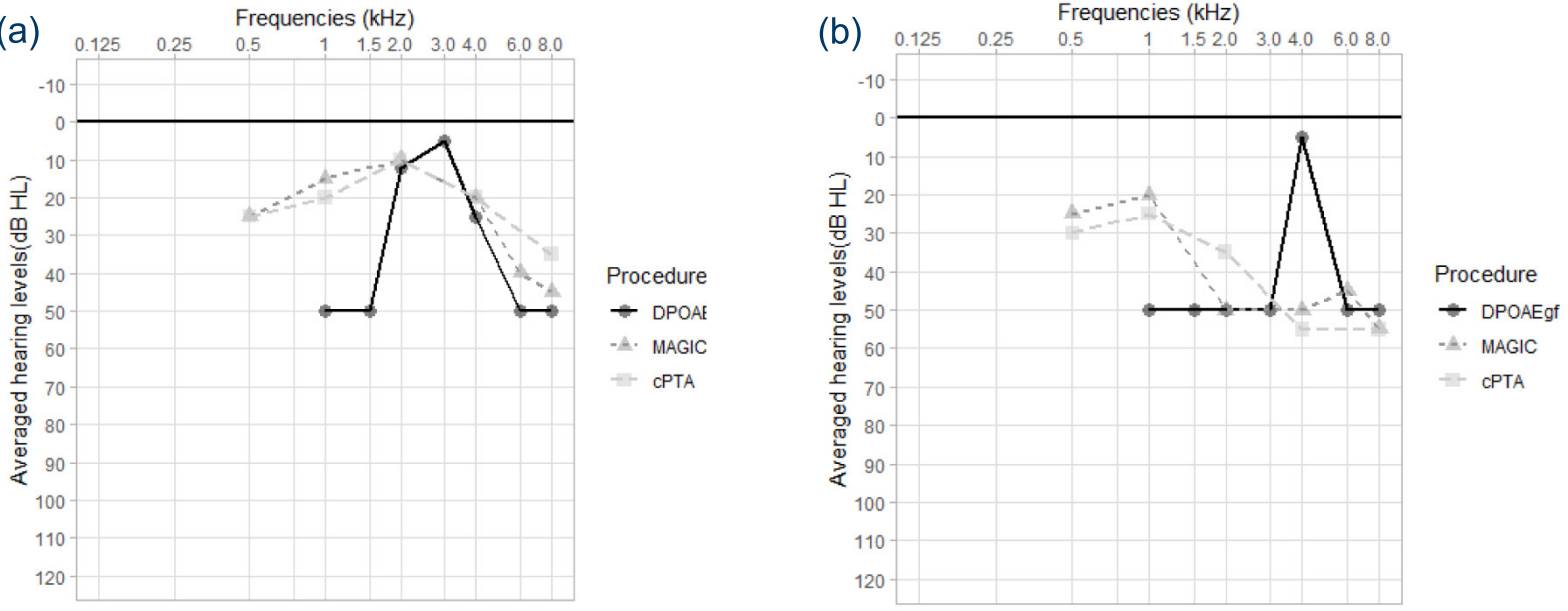
Example audiograms obtained with cPTA, MAGIC and DPOAEgf as representative of poor fit between PTA and DPOAEgf, shown at (a) lower and (b) higher EDPTs for individual frequencies.

Despite moderate‐to‐high correlations of EDPTs with PTA thresholds for group data (Boege and Janssen 2002), individual comparisons may show considerable deviations (Hoth et al. 2015). Our study findings agreed with this, in that despite high correlation (*Spearman's ρ* = 0.76, *p* < 0.001), DPOAE amplitudes and thus EDPT values for individual frequencies occasionally deviated considerably upward or downward from those of adjacent frequencies or from LPTATs (Figure 4).

Potential reasons for this variation are interferences of distortions and reflections, which can occur at different locations of the cochlea (Hoth et al. 2015). They cause DP‐grams and EDPTs measured at high frequency resolution to have a distinct fine structure with peaks and valleys associated with amplitude differences of up to 30–40 dB (Dhar et al. 2002; Talmadge et al. 1998, 1999, 2000). Although these fluctuations could be partly mitigated by lower‐frequency resolution or smoothing, we chose a fine‐grained measurement approach to better reflect the method's performance under real‐world testing conditions. The DPOAE obtained in the ear canal can be regarded as the vector sum of the (real and imaginary or primary and secondary) parts of the generator (distortion) and reflection components (Kalluri and Shera 2001; Knight and Kemp 2000; Shera and Guinan 1999; Talmadge et al. 1999). Constructive and destructive interactions of the two components lead to the formation of minima and maxima in DPOAEs (Naghibolhosseini 2023), the occurrence and amplitudes of which depend on factors such as stimulus levels, the frequency ratio *f*_2_/*f*_1_, hearing thresholds and age (Johnson and Baranowski 2012; Mauermann et al. 1999; Wagner et al. 2008). They can be smoothed by specific suppression paradigms (Heitmann et al. 1998) or with the help of pulse DPOAEs (Dalhoff et al. 2013; Zelle et al. 2016), which are not yet in clinical application but show good test–retest reliability particularly in the frequency range of 1–2 kHz (Bader et al. 2021). In the Sentiero, a frequency modulation procedure (FMDPOAE) provides some, though not complete, smoothing of the DPOAE fine structure to about 1% by modulating the stimuli at a frequency typically around 100 Hz and recording the resulting DPOAE response following the modulated 2*f*_1_–*f*_2_. If EDPTs or DPOAEgf are measured at only a few frequencies at large intervals, i.e., whole or half octaves as in our study, the fine structure is still not sufficiently resolved and some uncertainty remains regarding the location of each individual measurement point on a peak or in a valley (Figure 4). False‐negative or false‐positive DPOAE results in individual frequencies may thus result from the primary and secondary components interfering ‘destructively’ or ‘constructively.’ Even when fine structure is removed from DPOAEs, there can still be large variations in the predictions of individual thresholds using DPOAEgf (e.g., Zelle et al. 2017).

Further potential limitations of the study include the following: The test environment was not ideal in terms of ambient noise level, which may have influenced the quality of the DPOAE measurements. While generally quiet, the setting did not reach the low‐noise standards of a clinical environment. Most of the background noise originated from the participants themselves. As reported by Gorga et al. (1997), Gorga et al. (2000) and Gorga et al. (2003), the accuracy of the estimated hearing thresholds derived by extrapolation of I/O functions is typically higher at 2.0, 3.0 and 4.0 kHz than at higher and lower frequencies. This may in part be due to generally lower noise floors at these mid frequencies, which improve the reliability of DPOAE detection and threshold estimation. Moreover, Scheperle et al. (2008) demonstrated that standing waves in the ear canal, along with variations in probe insertion depth during in‐ear calibration, contribute to greater uncertainty in DPOAE measurements above 4 kHz. This may also partly account for the increased deviation of EDPTs from LPTATs at 6 kHz observed in our results.

## 5 Conclusions

The use of DPOAEgfs measured using mobile instrumentation has proven to be a feasible, quick and acceptably accurate alternative to hearing threshold estimation using PTA in the context of a universal programme of hearing screening, diagnosis, therapy and therapy monitoring for people with intellectual disabilities in their living environment, especially in cases where subjective tone audiometry is not possible or only possible to a limited extent. This is especially true where an average of four frequencies between 1.0 and 6.0 kHz is evaluated rather than single frequencies. The practical application of the DPOAEgf method in a large cohort study will provide further insights.

## Ethics Statement

The manuscript reports clinical observations on human subjects. The study was approved by the Institutional Ethics Review Board of the Ruhr University of Bochum (Approval Number 17‐6186).

## Conflicts of Interest

The authors declared no conflicts of interest.

## Supporting information


**Data S1:** Supplementary information.

**Figure S1:** Individual differences between LPTAT and EDPT for the frequencies 1.0, 2.0, 4.0 and 6.0 kHz for right (a) and left (b) ears.

## Data Availability

The data that support the findings of this study are available from the corresponding author upon reasonable request.
