# Supplementary figures and images for: Hearing Threshold Estimation With Distortion Product Otoacoustic Emission Growth Functions in People With Intellectual Disabilities in an Outreach Setting
Source: J Intellect Disabil Res. 2025 Sep 21;69(12):1474–85. doi: 10.1111/jir.70043 (PMC12580478; doi:10.1111/jir.70043)

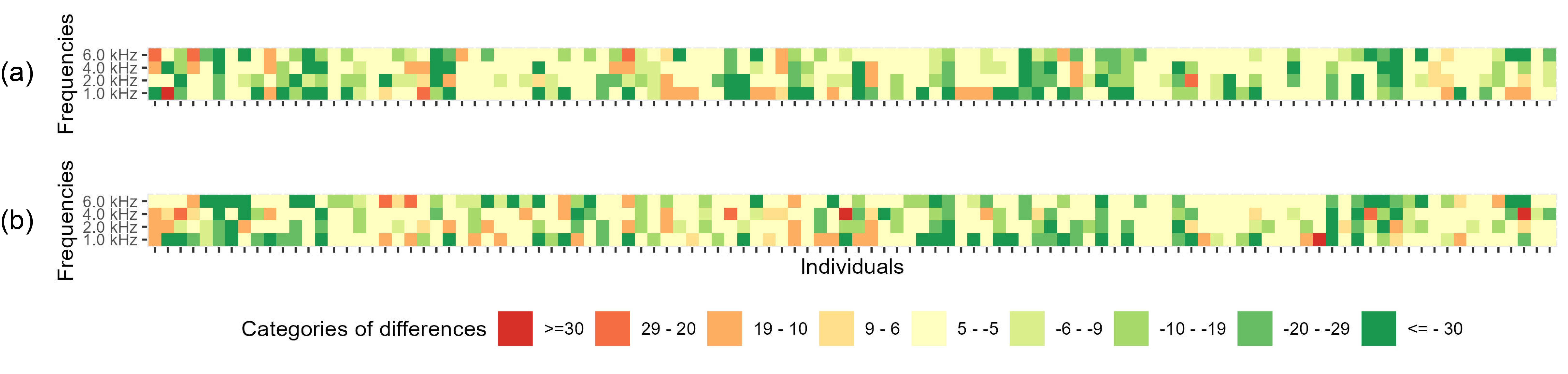

Supplement: Supplementary file 2 — Figure S1: Individual differences between LPTAT and EDPT for the frequencies 1.0, 2.0, 4.0 and 6.0 kHz for right (a) and left (b) ears. [file JIR-69-1474-s001.png]
